# Adrenal incidentalomas, cortisol secretion and cancer: is there a real crosstalk?

**DOI:** 10.3389/fendo.2023.1335202

**Published:** 2024-01-08

**Authors:** Aura D. Herrera-Martínez, Ángel Rebollo Román, Eider Pascual Corrales, Cindy Idrobo, Paola Parra Ramírez, Patricia Martín Rojas, Cristina Robles Lázaro, Marta Araujo-Castro

**Affiliations:** ^1^ Endocrinology & Nutrition Department, Hospital Reina Sofia, Córdoba, Spain; ^2^ Maimonides Institute for Biomedical Research of Cordoba (IMIBIC), Córdoba, Spain; ^3^ Endocrinology & Nutrition Department, Hospital Universitario Ramón y Cajal, Madrid, Spain; ^4^ Instituto de Investigación Biomédica Ramón y Cajal (IRYCIS), Madrid, Spain; ^5^ Endocrinology & Nutrition Department, Hospital La Paz, Madrid, Spain; ^6^ Endocrinology & Nutrition Department, Hospital de Salamanca, Salamanca, Spain

**Keywords:** cortisol, adrenal incidentaloma, cancer, mortality, mild autonomous cortisol secretion

## Abstract

**Background:**

Cortisol has immunomodulatory effects that increase the risk and evolution of several diseases. Cancer is characterized by a proinflammatory state in which cells exert impaired function and proliferation. The relation between cortisol secretion and increased risk of malignant neoplasm, or their behavior, has not been fully elucidated.

**Aim:**

To determine the relation between cortisol secretion and the prevalence and clinical outcome of malignant neoplasms in patients with adrenal incidentalomas (AIs).

**Methods:**

Multicenter retrospective study that included 935 patients with AIs. Cortisol secretion was defined by a cortisol post-dexamethasone suppression test > 1.8 µg/dL, and nonfunctioning AIs (NFAIs) as a value ≤ 1.8 µg/dL.

**Results:**

Cortisol secretion was evident in 30.8% of the patients and cancer in 23.6% (especially breast, colorectal, prostate and thyroid cancer). No differences in the cancer prevalence were found between patients with cortisol secretion and NFAIs (63.6% vs. 63.4%, p=0.10). After adjusting by age, cortisol secretion was not associated with the presence of cancer (OR 1.29, CI 0.93–1.78). However, cortisol secretion was significantly associated with stage IV of cancer at diagnosis (OR 2.68, CI 1.19– 6.00) and mortality (OR 3.2, CI 1.28- 7.97). Patients with NFAI and breast cancer required treatment with chemo- and radio-therapy more frequently that patients with cortisol secreting AI (90% vs 10% and 92.9% vs 7.1% respectively, p<0.05), similarly patients with prostate cancer required radiotherapy more frequently (90.9% vs 9.1%, p=0.05); also, patients with colorectal cancer and NFAI, tended to require chemotherapy more frequently(76.5% vs 23.5%, p=0.06).

**Conclusion:**

Cortisol secretion does not increase the risk of malignant neoplasm, but it affects its clinical course, treatment requirements and mortality, leading to a worst prognosis and higher mortality when compared with patients with NFAIs.

## Introduction

Hormonal evaluation of patients with adrenal incidentalomas (AIs) is one of the most common consultation reasons for clinical endocrinologists. An appropriate hormonal evaluation is necessary, since up to 50% can present with excess hormone secretion (1, 2). Specifically, cortisol secretion is the most common type of hypersecretion (3).

Mild and overt cortisol secretion have been associated with increased morbidity and mortality (3). Even mild autonomous cortisol secretion (MACS) requires individualized management depending on the associated comorbidities and grade of control of them and the epidemiological and clinical characteristics of the patients (1, 2), since increased cardiovascular events and mortality have been described also in these patients (4, 5). Specifically, serum cortisol >1.8 mcg/dL after midnight 1 mg dexamethasone suppression test (DST) is associated with increased mortality due to infections and cardiovascular disease (6, 7). All these studies led to the conclusion that prolonged exposure to elevated cortisol levels can produce maladaptive responses that conditionate not only the development of metabolic complications, but also the presence of other complications related to altered systemic inflammation and stress.

Additionally, recent publications have suggested that cortisol might play a role in cancer development. Cortisol produces immunosuppression, reducing immunosurveillance of early-stage cancer and facilitating their immune escape and acquisition of further oncogenic mutations (8, 9). Despite cortisol is essential for initial inflammatory response, its chronic secretion has been associated with cortisol dysfunction and increased inflammation (10). Furthermore, cancer itself can also produce changes in systemic inflammation, inducing changes in the activation of the hypothalamus-pituitary-adrenal axis (HPA) and disturbing the appropriate stress-induced cortisol secretion (11, 12).

Moreover, cortisol is associated with insulin resistance and obesity, which are closely related with increased risk for a range of malignancies (13–15). Hyperinsulinism is associated with increased insulin-like growth factor I (IGF-I), which seems to have a role in tumor initiation and progression in insulin-resistant patients. Insulin and IGF-I inhibit the hepatic synthesis of sex-hormone binding globulin (SHBG), additionally, both hormones stimulate estrogen secretion in the ovary, which can promote cellular proliferation and inhibit apoptosis of breast and endometrium cells (16). Hyperinsulinism can also increase the production of reactive oxygen species (ROS), which can produce DNA damage and increase the risk of mutagenesis and carcinogenesis (16). Finally, adipocytes can release several interleukins that promote systemic inflammation which can result in a favorable environment for tumor growth (14). Other publications suggest an additional mechanism that links MACS and cancer, which is closely related to stress levels; specifically, several studies have reported that chronic exposure to physiological stress also increases cancer risk (17). In stressful situations, chronic low-grade inflammation and declined immune surveillance are present, both induced by stress and also related with cancer development and progression (18). Despite this, some reports are still contradictory (19).

All these mechanisms are synergistic and suggest that cortisol may affect the prevalence of cancer, especially of some specific cancer subtypes. In this context, we aimed to determine the prevalence of cancer in patients with AIs and compare their clinical evolution according to the presence or not of cortisol secretion. Finally, we evaluated the clinical evolution and treatment of the most prevalent cancer subtypes in our cohort: colorectal, breast, prostate, thyroid, and neuroendocrine tumors.

## Materials and methods

### Patients

This study was approved by the Ethics Committee of the Ramón y Cajal University Hospital (Madrid, Spain), which was conducted in accordance with the Declaration of Helsinki and according to national and international guidelines. This is a multicenter retrospective study, wherein patients with AIs of four Spanish Hospitals were included [Reina Sofía University Hospital (Córdoba), Ramón y Cajal University Hospital (Madrid), La Paz University Hospital (Madrid) and Salamanca University hospital (Salamanca)]. Nine hundred thirty-five patients with AIs were included. Clinical records were used to collect full medical history. All patients were managed following available guidelines and recommendations (1, 2). The prevalence of cancer was evaluated in each case, as well as its stage at diagnosis and clinical management. Additional information about persistence disease and mortality were collected. Clinical and surgical management of the malignant neoplasms were performed by the corresponding specialists according to the specific clinical guidelines.

### Hormonal and imaging data

Only patients with adrenal incidentalomas that presented imaging-based characteristics of adenomas were included (1, 2, 20). Hormonal study of the AI was performed as indicated in the current clinical guidelines (1, 2, 20). Serum baseline morning cortisol, adrenocorticotropic hormone (ACTH), dehydroepiandrostendione sulphate (DHEAS), 24h urinary free cortisol (UFC) and 1 mg dexamethasone suppression test (DST) were evaluated in this study. Patients were classified in two main groups: NFAI when DST was <1.8 mcg/dL and cortisol secretion when DST were above 1.8 µg/dL. In this second group we included patients with mild autonomous cortisol secretion (with normal UFC and nonspecific clinical data of overt Cushing syndrome) and patients with overt Cushing´s syndrome (high UFC and specific clinical data of hypercortisolism) (7).

### Statistical analysis

Continuous variables were expressed as median with interquartile range and categorical variables were described as proportions. For missing data and specific group analysis, the absolute number has been also expressed in brackets.

Between-group comparisons were analyzed by the Mann–Whitney U test (nonparametric data). Paired analysis was performed by Student t (parametric data) or Wilcoxon test (nonparametric data). Chi-squared test was used to compare categorical data. Statistical analyses were performed using SPSS statistical software version 20, and Graph Pad Prism version 9. P-values <0.05 were considered statistically significant.

## Results

### Malignant neoplasms, nonfunctioning and cortisol secreting adrenal incidentalomas

Nine hundred thirty-five patients were included, 53.4% males and with a median age at the diagnosis of the AI of 62 y-old. About a half of patients were active or former smokers (27.3% and 39.1% respectively). In 69.2% of patients, AI were nonfunctioning, while mild or overt cortisol secretion was observed in 30.8% of patients (n=288). The prevalence of metabolic comorbidities was similar in nonfunctioning and cortisol secreting AIs. Detailed characteristics of the cohort are depicted in **Table 1**.

**Table 1:** General characteristics of all included patients.

Characteristic	All patients (n=935)	Nonfunctioning AI (n=647)	Cortisol secreting AI (n=288)	*p*
Age at diagnosis	62.4 (54-71)	46 (39 – 60)	57 (54 – 68)	0.02
Sex (%male/%female)	53.4/46.6	52.9/47.1	54.4/45.6	0.07
Tobacco exposure				
Active smoker	27.3 (54/198)	63 (34/54)	37 (20/54)	0.20
Former smoker	39.1 (219/560)	68.5 (150/219)	31.5 (69/219)	0.61
Complications				
Hypertension	54.9 (511/930)	64.8 (331/511)	35.2(180/511)	0.33
Diabetes	24.9 (230/925)	64.8 (149/230)	35.2 (81/230)	0.06
Dyslipidemia	47.9 (443/925)	68.4 (303/443)	31.6 (140/243)	0.33
Cardiovascular complications	2.1 (19/926)	63.2 (12/19)	36.8 (7/19)	0.37
Cerebrovascular complications	10.8 (100/922)	65 (65/100)	35 (35/100)	0.18
Obesity	40.9 (300/734)	69.7 (209/300)	30.3 (91/300)	0.23
Other neoplasms	23.6 (221/935)	64.7 (143/221)	35.3 (78/221)	0.11
Age at diagnosis	63.5 (55-72)	63.4 (55-71)	63.6 (54-71)	0.10
Location				
Colorectal	4.1 (38/935)	65.8 (25/38)	34.2 (13/38)	0.72
Hematological cancer	1.4 (13/935)	46.2 (6/13)	53.8 (7/13)	0.12
Breast	4.3 (40/935)	67.5 (27/40)	32.5 (13/40)	0.86
Prostate	2.6 (24/935)	75 (18/24)	25 (6/24)	0.66
Lung	1.3 (12/935)	66.7 (8/12)	33.3 (4/12)	0.53
Thyroid	2.6 (24/935)	58.3 (14/24)	41.7 (10/24)	0.27
Pancreas	0.3 (3/935)	66.7 (2/3)	33.3 (1/3)	0.67
Neuroendocrine tumors	1.8 (17/935)	58.8 (10/17)	41.2 (7/17)	0.43
Bladder	1 (9/935)	55.6 (5/9)	44.4 (4/9)	0.40
Kidney	1.1 (10/935)	90 (9/10)	1 (1/10)	0.07
Cervix	0.4 (4/935)	25 (1/4)	75 (3/4)	0.12
Uterus	0.1 (1/935)	100 (1/1)	0	
Ovary	0.3 (3/935)	66.7 (2/3)	33.3 (1/3)	0.72
Endometrium	0.7 (7/935)	71.4 (5/7)	28.6 (2/7)	0.52
Skin	0.6 (6/935)	66.7 (4/6)	33.3 (2/6)	0.64
Soft tissue	1.1 (10/935)	70 (7/10)	30 (3/10)	0.50
Bone	0.1 (1/935)	100 (1/1)	0	
Head and neck cancer	1.4 (13/935)	62.9 (9/13)	30.8 (4/13)	0.48
Central Nervous system	0.6 (6/935)	66.7 (4/6)	33.3 (2/6)	0.64
Liver	0.2 (2/935)	50 (1/2)	50 (1/2)	0.59
Stomach	0.9 (8/935)	50 (4/8)	50 (4/8)	0.29
Stage at diagnosis				
I	48.8 (78/160)	74.8 (58/78)	25.6 (20/78)	0.05
II	16.9 (27/160)	70.4 (19/27)	29.6 (8/27)	0.46
III	14.4 (23/160)	65.2 (15/23)	34.8 (8/23)	0.49
IV	20 (32/160)	50 (16/32)	50 (16/32)	0.01
Treatment				
Surgery	80.6 (175/217)	64.6 (113/175)	35.4 (62/175)	0.47
Chemotherapy	31.4 (65/207)	69.2 (45/65)	0.8 (20/65)	0.35
Radiotherapy	30.3 (63/208)	71.4 (45/63)	28.6 (18/63)	0.21
Others	19.9 (30/66)	70(21/30)	30 (9/30)	0.51
Persistent disease	34.5 (78/226)	65.4 (51/78)	34.6 (27/78)	0.51
Mortality	2.1 (20/935)	40 (8/20)	60 (12/20)	0.006

AI, adrenal incidentaloma.

In the whole cohort, 23.6% presented a malignant neoplasm; among them, 64% were observed in patients with NFAIs (143 cases of 647, 22.2% of its group) and 35.3% occurred in the cortisol secreting group (78 cases of 288: 27%), with no significant difference between groups. Breast, colorectal, prostate and thyroid were the most prevalent neoplasms in the cohort, but up to twenty different types of cancer were observed. The distribution of each type of cancer was not statistically different in patients with nonfunctioning and cortisol secreting AIs except for kidney cancer, which tended to be more prevalent in patients with NFAIs (p=0.06; **Table 1**). After adjusting by age, cortisol secretion was not associated with the presence of cancer (OR 1.29, CI 0.93– 1.78; **Table 2**).

**Table 2:** Multivariate logistic regression for the presence of cancer in patients with cortisol secreting AI adjusted by age.

Variable		OR	CI	*p*
Tumor location	All types of cancer	1.29	0.93 – 1.78	0.12
	Breast cancer*	1.05	0.50 – 2.21	0.89
	Colorectal cancer*	1.11	0.56 – 2.24	0.76
	Prostate cancer	0.63	0.24 – 1.63	0.34
	Thyroid cancer	1.55	0.55 – 3.57	0.29
	Neuroendocrine tumors	1.84	0.68 – 5.00	0.23
	Hematologic cancer	2.31	0.76 – 7.04	0.14
	Lung	1.01	0.30 – 3.43	0.98
	Pancreas	1.14	0.10 – 1.27	0.91
	Bladder	1.35	0.32 – 5.73	0.68
	Cervix	6.12	0.59 – 63.36	0.12
	Kidney	0.16	0.02 – 1.35	0.09
	Ovary	1.65	0.09 – 30.34	0.73
	Central nervous system	0.91	0.15 – 5.32	0.91
	Skin	0.77	0.13 – 4.70	0.78
	Head and neck cancer	0.76	2.21 - 2.74	0.67
	Soft tissue	0.74	0.18 – 3.12	0.68
	Endometrium	0.69	0.13 – 3.90	0.69
	Liver	1.69	0.01 – 8.6	0.71
	Gastric	1.87	0.43 - 0.16	0.40
Stage at diagnosis	Stage I	0.53	0.27 – 1.04	0.06
	Stage IV	2.68	1.19 – 6.00	0.01
Persistent disease		0.98	0.55 – 1.45	0.93
Mortality		3.20	1.28 - 7.97	0.03

*also adjusted by obesity

A higher proportion of cancer in stage I was observed at diagnosis in patients with nonfunctioning AIs (74.8% of all stage I neoplasms) compared with cortisol secreting AIs (25.6% of all stage I neoplasms, p=0.05); furthermore, in this last group, stage IV was more prevalent (5.6%) than in the nonfunctioning group (2.5%, p<0.01), despite 16 cases were observed in each group (50% of all the stage IV patienst). After adjusting by age, cortisol secretion was significantly associated with the presence of stage IV of disease at diagnosis (OR 2.68, CI 1.19– 6.00) but not stage I (OR 0.53, CI 0.27– 1.04).

Persistence of malignant disease was similar in both groups, but mortality was increased in patients with cortisol secreting AIs (p<0.01). After adjusting by age, cortisol secretion was still significantly associated with mortality (OR 3.2, CI 1.28 -7.97) but not with persistent disease (OR 0.98, CI 0.55 –1.45; **Table 2**).

**Figure 1:**
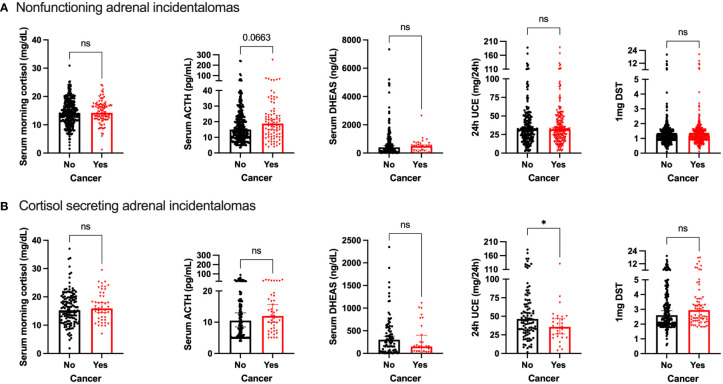
Comparison of hormone profile in patients with and without cancer in **(A)** nonfunctioning adrenal incidentalomas; **(B)** cortisol secreting adrenal incidentalomas. ns, non-significant; *p<0.05.

In patients with NFAIs, serum ACTH levels tended to be increased in patients with cancer (median 18.7 pg/mL, IQR: 11-29; p=0.06) compared with patients without cancer (median 14 pg/mL, IQR: 10-23); but baseline cortisol levels were similar in both groups. There were no differences in serum DHEA-S, DST or UFC between both groups (**Figure 1A**).

In contrast, patients with cancer and cortisol secreting AIs presented with lower UFC compared with patients without cancer (median 35.7 mg/24h, IQR: 22.5-49.2 vs 46.4 mg/24h, IQR: 31.3 – 78.8 respectively; p<0.05; **Figure 1B**). There were no differences in serum cortisol, ACTH, DHEAS or DST between both groups (**Figure 1B**).

### Breast cancer

Forty patients presented with breast cancer. In these patients, the diagnosis of AI was made at a younger age in those with cortisol secreting AI compared with patients with NFAI (58.9 vs 67.3 years, respectively, p=0.06). Remarkably, the prevalence of diabetes and obesity was statistically higher in the NFAI group than in the cortisol secreting group (100% vs 0%, p=0.04 and 91.7% vs8.3%, p=0.02 respectively). Although, the stage I of disease tended to be more prevalent in patients with NFAI (80% vs 20%), and stage III (33.3% vs 66.7%) was in patients with cortisol secreting AI, these results were non-statically significant. Regarding therapeutic options, patients with cortisol-secreting AI required radiotherapy less frequently (7.1% vs 92.9%; p<0.01) and tended to require less chemotherapy (10% vs 90%; p=0.07) than patients with NFAIs. Persistent disease was similar in both groups. Detailed results are depicted in **Table 3**. After adjusting by age and the presence of obesity, cortisol secretion was not associated with the presence of breast cancer (OR 1.05, CI 0.50– 2.21; **Table 2**).

**Table 3:** Characteristics of patients with breast cancer and adrenal incidentaloma.

Characteristic	All patients (n=40)	Nonfunctioning AI(n=27)	Cortisol secreting AI (n=13)	*p*
Age at diagnosis of AI	63.9 (57-70)	67.3 (59.6-71)	58.9 (53-61.5)	0.06
Age at cancer diagnosis	58.9(50 – 67)	65.4 (50 -70)	52 (51-58)	0.15
Tobacco exposure				
Active smoker	25 (3/8)	50 (1/2)	50 (1/2)	0.79
Former smoker	25 (5/20)	60 (2/5)	40 (3/5)	0.30
Complications				
Hypertension	42.5 (17/40)	70.6 (12/17)	29.4(5/17)	0.49
Diabetes	17.5 (7/40)	100 (7/7)	0	0.04
Dyslipidemia	65 (26/40)	61.5 (16/26)	38.5 (10/26)	0.33
Cardiovascular complications	0	0	0	0.37
Cerebrovascular complications	12.5 (5/40)	60 (3/5)	40 (2/5)	0.53
Obesity	36.4 (12/33)	91.7 (11/12)	8.3 (1/12)	0.02
Stage at diagnosis				
I	55.6 (10/18)	80 (8/10)	20 (2/10)	0.38
II	22.2 (4/18)	75 (3/4)	25 (1/4)	0.71
III	16.7 (3/18)	33.3 (1/3)	66.7 (2/3)	0.17
IV	5.6 (1/18)	100 (1/1)	0	0.20
Treatment				
Surgery	100 (29/29)	69 (20/29)	31 (9/29)	0.47
Chemotherapy	35.9 (10/28)	90 (9/10)	10 (1/10)	0.07
Radiotherapy	50 (14/28)	92.9 (13/14)	7.1 (1/14)	0.006
Others	42.5 17/40)	70.6 (12/17)	30.4 (5/17)	0.86
Persistent disease	18.2 (6/33)	66.7 (4/6)	33.3 (2/6)	0.53

AI, adrenal incidentaloma.

### Colorectal cancer

Colorectal cancer was diagnosed in 38 patients. The age at diagnosis of AI and colorectal cancer was similar in patients with and without cortisol secreting AI. A higher proportion of patients with hypertension was observed in the NFAI group (53.8% vs 46.2%; p<0.05), while other metabolic comorbidities, including obesity, were similar in both groups. Similar proportions of patients with stage I and II colorectal cancer were observed at diagnosis in both groups, but the percentage of patients with stage III was higher in the group of NFAIs (90% vs 10%, p<0.05). Regarding therapy, patients with NFAI tended to require treatment with chemotherapy more frequently than the cortisol secreting group (76.5% vs 23.5%, p=0.06). Other therapeutic options, persistent disease and mortality were comparable in both groups (**Table 4**). After adjusting by age and the presence of obesity, cortisol secretion was not associated with the presence of colorectal cancer (OR 1.11, CI 0.55– 2.24; **Table 2**).

**Table 4:** Characteristics of patients with colorectal cancer and adrenal incidentaloma.

Characteristic	All patients (n=38)	Nonfunctioning AI(n=25)	Cortisol secreting AI (n=13)	*p*
Age at diagnosis of AI	69.5 (63 -73)	71.1 (65.6 -73)	65.8 (59-72)	0.22
Age at cancer diagnosis	67(59 – 75)	66.8 (60 - 76)	68.7 (59-74)	0.98
Tobacco exposure				
Active smoker	2.6 (1/7)	100 (1/1)	0	0.57
Former smoker	15.8 (6/20)	66.7 (4/6)	33.3 (2/6)	0.43
Metabolic Complications				
Hypertension	68.4 (26/38)	53.8 (14/26)	46.2 (12/26)	0.02
Diabetes	42.1 (16/38)	50 (8/16)	50 (8/16)	0.08
Dyslipidemia	65.8 (25/38)	64 (16/25)	36 (9/25)	0.52
Cardiovascular complications	0	0	0	0.37
Cerebrovascular complications	31.6 (12/38)	66.7 (8/12)	33.3 (4/12)	0.62
Obesity	45.5 (15/35)	60 (9/15)	40 (6/15)	0.49
Stage at diagnosis				
I	17.9 (5/28)	60 (3/5)	40 (2/5)	0.60
II	25 (7/28)	42.9 (3/7)	57.1 (4/7)	0.18
III	35.7 (10/28)	90 (9/10)	10 (1/10)	0.04
IV	21.4 (6/28)	50 (3/6)	50 (3/6)	0.36
Treatment				
Surgery	96.7 (29/30)	65.5 (19/29)	34.5 (10/29)	0.37
Chemotherapy	58.6 (17/29)	76.5 (13/17)	23.5 (4/17)	0.06
Radiotherapy	31 (9/29)	66.7 (6/9)	33.3 (3/9)	0.53
Others	5.3 (2/38)	50 (1/2)	50 (1/2)	0.49
Persistent disease	29 (9/31)	77.7 (7/9)	22.2 (2/9)	0.29
Mortality	2.6 (1/38)	0	100 (1/1)	0.34

AI, adrenal incidentaloma.

### Prostate cancer

In this cohort, 24 males presented with prostate cancer. As in colorectal cancer, a higher proportion of hypertension was diagnosed in patients with NFAI (53.8% vs 46.2%; p<0.05); while age and other metabolic comorbidities were not statistically significant different in both groups. More patients with grade 2 tumors (Gleason 7) were observed in the group of NFAI compared with cortisol-secreting AI (90% vs 10%; p<0.05). Additionally, surgery for prostate cancer was more common in patients with cortisol-secreting AI (54.5%) than in patients with NFAI (45.5%; p<0.01). Furthermore, patients of this last group tended to require treatment with radiotherapy more frequently (91.7% vs 8.3%; p=0.05). Persistent disease also tended to be also more frequent in this group (p=0.09; **Table 5**). After adjusting by age, cortisol secretion was not associated with the presence of prostate cancer (OR 0.63, CI 0.24– 1.63; **Table 2**).

**Table 5:** Characteristics of patients with prostate cancer and adrenal incidentaloma.

Characteristic	All patients (n=24)	Nonfunctioning AI(n=18)	Cortisol secreting AI (n=6)	*p*
Age at diagnosis of AI	61 (65-77)	72.7 (69-77)	65.6 (61-71)	0.25
Age at cancer diagnosis	67 (61 – 74)	71 (60 -75)	64 (62-64)	0.52
Tobacco exposure				
Active smoker	50 (2/4)	100 (1/1)	0	0.57
Former smoker	46.2 (6/13)	66.7 (4/6)	33.3 (2/6)	0.43
Metabolic Complications				
Hypertension	79.2 (19/24)	53.8 (14/26)	46.2 (12/26)	0.02
Diabetes	29.2 (7/24)	50 (8/16)	50 (8/16)	0.08
Dyslipidemia	50 (12/24)	64 (16/25)	36 (9/25)	0.52
Cardiovascular complications	0	0	0	0.37
Cerebrovascular complications	29.2 (7/24)	66.7 (8/12)	33.3 (4/12)	0.62
Obesity	21.1 (4/19)	60 (9/15)	40 (6/15)	0.49
Stage at diagnosis				
Gleason X	54.2 (13/24)	60 (3/5)	40 (2/5)	0.60
G1 (Gleason <=6)	12.5 (3/24)	42.9 (3/7)	57.1 (4/7)	0.18
G2 (Gleason 7)	12.5 (3/24)	90 (9/10)	10 (1/10)	0.04
G3 (Gleason 8-10)	20.8 (5/24)	50 (3/6)	50 (3/6)	0.36
Treatment				
Surgery	55 (11/24)	45.5 (5/11)	54.5 (6/11)	0.01
Chemotherapy	0	0	0	
Radiotherapy	60 (12/20)	91.7 (11/12)	8.3 (1/12)	0.05
Others	45.8 (11/24)	90.9 (10/11)	9.1 (1/11)	0.23
Persistent disease	28.6 (6/21)	100 (6/6)	0	0.09
Mortality	8.3 (2/24)	78.9 (15/19)	21.1 (4/19)	0.36

AI, adrenal incidentaloma; G, Gleason score.

### Thyroid cancer

Thyroid cancer was diagnosed in 24 patients. Cortisol secreting AIs were diagnosed in older patients with thyroid cancer (median age 72 y-old) than in patients with NFAIs (median age 62 y-old; p<0.05). We did not observe significant differences among metabolic comorbidities, stage of thyroid cancer at diagnosis, therapeutic management and persistent disease in both groups (**Table 6**). After adjusting by age, cortisol secretion was not associated with the presence of thyroid cancer (OR 1.56, CI 0.55– 3.57; **Table 2**).

**Table 6:** Characteristics of patients with thyroid cancer and adrenal incidentaloma.

Characteristic	All patients (n=24)	Nonfunctioning AI(n=14)	Cortisol secreting AI (n=10)	*p*
Age at diagnosis of AI	65 (59-73)	62.4 (57-67)	72.1 (65-76)	0.02
Age at cancer diagnosis	57 (48 – 64)	56.6 (44 -65)	57.2 (49-63)	0.10
Tobacco exposure				
Active smoker	0	0	0	
Former smoker	20 (3/15)	66.7 (2/3)	33.3 (1/3)	0.65
Metabolic Complications				
Hypertension	58.3 (14/24)	50 (7/14)	50 (7/14)	0.29
Diabetes	25 (6/24)	33.3 (2/6)	66.7 (4/6)	0.17
Dyslipidemia	37.5 (9/24)	44.5 (4/9)	55.6 (5/9)	0.26
Cardiovascular complications	0	0	0	0.37
Cerebrovascular complications	20.8 (5/24)	60 (3/5)	40 (2/5)	0.66
Obesity	40 (6/15)	83.3 (5/6)	16.7 (1/6)	0.16
Stage at diagnosis				
I	40 (6/15)	33.3 (2/6)	66.7 (4/6)	0.37
II	20 (3/15)	66.7 (2/3)	33.3 (1/3)	0.44
III	13.3 (2/15)	50 (1/2)	50 (1/2)	0.73
IV	26.7 (4/15)	50 (2/4)	50 (2/4)	0.66
Treatment				
Surgery	100 (18/18)	50 (9/18)	50 (9/18)	–
Chemotherapy	18.8 (3/16)	100 (3/3)	0	0.10
Radiotherapy	31.3 (5/16)	60 (3/8)	40 (2/8)	0.50
Others	66.7 (16/24)	50 (8/16)	50 (8/16)	0.35
Persistent disease	50 (9/18)	55.6 (5/9)	44.4 (4/9)	0.50
Mortality	4.2 (1/24)	100 (1/1)	0	0.58

AI, adrenal incidentaloma.

### Neuroendocrine tumors

There were no significant differences between age and metabolic comorbidities in patients with neuroendocrine tumors (NETs) and cortisol secreting and NFAIs (**Table 6**). Despite more patients with NET stage 1 were diagnosed in patients with NFAIs (80% vs 20%, p=0.57), this difference was not statistically significant, furthermore, there were no significant differences in therapeutic management or persistence disease between both groups (**Table 7**). After adjusting by age, cortisol secretion was not associated with the presence of NETs (OR 1.837, CI 0.676 – 4.995; **Table 2**).

**Table 7:** Characteristics of patients with neuroendocrine tumors and adrenal incidentaloma.

Characteristic	All patients (n=17)	Nonfunctioning AI(n=10)	Cortisol secreting AI (n=7)	*p*
Age at diagnosis of AI	57.3 (49 -70)	58.8 (50-76)	55.7 (49 -59)	0.60
Age at cancer diagnosis	59 (52 – 67)	63.8 (54 -74)	58 (50 - 59)	0.18
Tobacco exposure				
Active smoker	20 (1/5)	0	100 (1/1)	0.40
Former smoker	12.5 (1/8)	100 (1/1)	0	0.50
Metabolic Complications				
Hypertension	70.6 (12/17)	58.3 (7/12)	41.7 (5/12)	0.68
Diabetes	41.2 (7/17)	71.4 (5/7)	28.6 (2/7)	0.35
Dyslipidemia	47.1 (8/17)	50 (4/8)	50 (4/8)	0.42
Cardiovascular complications	11.8 (2/17)	50 (1/2)	50 (1/2)	0.67
Cerebrovascular complications	5.9 (1/17)	0	100 (1/1)	0.41
Obesity	66.7 (8/12)	75 (6/8)	25 (2/8)	0.40
Stage at diagnosis				
I	45.5 (5/11)	80 (4/5)	20 (1/5)	0.57
II	9.1 (1/11)	100 (1/1)	0	0.72
III	27.3 (3/11)	33.3 (1/3)	66.7 (2/3)	0.15
IV	11.8 (2/11)	100 (2/2)	0	0.50
Treatment				
Surgery	64.3 (9/14)	44.4 (4/9)	55.6 (5/9)	0.06
Others	41.2 (7/17)	71.4 (5/7)	28.6 (2/7)	0.28
Persistent disease	55.7 (10/15)	80 (8/10)	20 (2/10)	0.16
Mortality	0	0	0	–

AI, adrenal incidentaloma.

## Discussion

Cortisol is the main glucocorticoid released from the adrenal cortex. It has many physiological effects in the human body, including regulation of stress response, metabolism, inflammatory response, and immune function (21). Probably due to its relevant role in several organs and human systems, its overproduction is related with several comorbidities and increased mortality (4, 5).

According to the International Agency for Research on Cancer, in 2020, 19.3 million new cancer cases (18.1 million excluding nonmelanoma skin cancer) and almost 10.0 million cancer deaths (9.9 million excluding nonmelanoma skin cancer) were estimated to occur worldwide (22). Currently, cancer diagnosis and treatment represent a challenge for clinicians and health systems in several countries. In this context, this study presents a large well-characterized cohort of patients with AIs and compares the prevalence (and outcome) of cancer in patients with NFAIs and cortisol secretion (mild autonomous cortisol secretion and overt Cushing syndrome). To the best of our knowledge, this is the first study focused on the evaluation of adrenal cortisol secretion and cancer in the real clinical practice.

Prevalence of cancer ranges 0.4-5.5% according to the World Health Organization. In our cohort it was higher, probably due to the fact that all patients underwent at least one abdominal imaging technique. Colorectal, breast, thyroid and prostate were the most prevalent malignant neoplasms in our sample, similar to worldwide statistics, in which female breast cancer is the most commonly diagnosed cancer, with an estimated 2.3 million new cases (11.7%), while colorectal and prostate cancers exhibit a prevalence of 10.0% and 7.3% respectively (22).

Cancer prevalence was not affected by cortisol secretion in our cohort, but we found a higher proportion of patients at an advanced stage of disease at diagnosis in the cortisol secreting AI group. This finding is in line with the hypothesis that cortisol produces immunosuppression and in consequence reduces immunosurveillance of early-stage cancer, facilitating immune escape and acquisition of further oncogenic mutations (8, 9), which might contribute to tumor progression but not to tumorigenesis.

Increased cortisol levels in Cushing’s disease have been associated with increased testosterone levels and decreased SHBG levels (23); elevated levels of serum estrogen and testosterone increase the risk of some types of cancer (including breast, endometrium, and prostate), additionally, the bioactive concentrations of estrogen and testosterone are influenced by SHBG (24). Additionally, there is complex feedback between estrogen and cortisol serum levels (25, 26). In this context, Larsson et al. (27) described that the association between cortisol levels and cancer is increased in patients with hormone-related subtypes. For example, previous studies have reported that genetically predicted plasma cortisol is associated with increased risk of endometrial cancer (OR 1.50, 95% CI 1.13–1.99) but not with other cancers (27). *In vitro* studies reported poorer prognosis in endometrial tumors with high expression of estrogen and glucocorticoids receptors (28). The prevalence of endometrium cancer in our cohort was too low to mark separate analysis. In contrast, other endocrine-related cancers such breast and prostate cancer were analyzed separately. Specifically, breast cancer tended to be increased in patients with cortisol secretion in our cohort but required less frequently systemic treatment with chemo- or radiotherapy. Some types of breast cancer are closely related to estrogen receptor expression. Obesity is also strongly associated with an increased risk of different cancer types including endometrial, colorectal and breast cancer (29), but remarkably, it was not increased in patients with breast cancer and cortisol secretion. Interestingly, its prevalence was similar in the whole cohort and all cancer subtypes (but breast cancer), suggesting the relevance of obesity on inflammation and tumorigenesis (14).

Elevated cortisol levels have been previously described in patients with prostate cancer (30, 31), despite this, a clear association between cortisol levels and tumorigenesis has not been stablished yet. In the group of males with cortisol secretion in our cohort, prostate cancer was more frequently surgically resected, patients required less frequently radiotherapy and more patients tended to be free of disease.

In this study, prevalence of colorectal cancer was not increased in patients with cortisol secretion, but metabolic comorbidities were increased. It has been described that some lifestyle habits including poor diet can activate HPAA axis, increasing cortisol levels and resulting in brain-gut axis disturbance and immune dysregulation in the gastrointestinal tract. This dysregulation could produce food digestion, absorption and an irritated/inflamed mucosal lining (32). This resulting mucosal inflammation also contributes to maintain elevated cortisol levels, with its subsequent effects disorganizing the immune system, which in turn cannot respond to persistent inflammation and could affect the clinical evolution of cancer in the gastrointestinal tract (33, 34). During the last years, some authors have reported a causal contribution of the microbiome to stress-related disorders (35), it has been associated with gut microbiota diversity (36) and even the use of some prebiotics has been associated to decreased cortisol levels in humans (37).

Interestingly, clinical characteristics of the patients with thyroid cancer and NETs, which are common neoplasms evaluated by endocrinologists, were not statistically different in patients with cortisol secretion and NFAIs.

Previous studies have described increased AIs and thyroid nodules in elderly patients, probably due to increased arteriopathy of the gland arteries (38), but there are no specific reports of cortisol secretion and thyroid cancer (apart from ACTH secretion in medullary thyroid cancer [39, 40)]. Furthermore, it is well-known that cortisol secretion due to ectopic ACTH secretion in NETs is associated with worse clinical outcomes (41, 42) but the effect of cortisol secretion with suppressed ACTH is not still understood.

Remarkably, cortisol secretion was significantly and independently associated with mortality in all patients after adjusting by age. This finding has been previously reflected in other cohorts (6, 43). This fact suggests that the presence of a malignant neoplasm could be also considered when a patient with AI is evaluated, specifically, medical or surgical treatment might also be considered since it might influence the clinical evolution or mortality of the patient. Additionally, antitumorigenic effects of steroidogenesis inhibitors have been also described (44, 45) and could provide an additional benefit to these patients.

This study has some limitations. First the retrospective nature of the study, which is accompanied by an intrinsic risk of bias and missing data; additionally, only the primary tumor location was considered and not the specific histologic characteristic of the tumor also, the number of participants with overt cortisol secretion was not big enough to make a third group of comparison. But probably, the most important limitation is the reduced number of malignant neoplasms, especially in some types of cancer, which could have led to non-significant results in clinically relevant variables including progressive disease and mortality. Also due to the number of malignant neoplasms, results were not analyzed separately according sex; it is well-known that tumor behavior could present with sex-related differences, and this factor should be also considered. In contrast, this study has several strengths: it is a large cohort of well-characterized patients with AIs, and not only the prevalence, but also disease extension at diagnosis and clinical evolution were considered. Moreover, persistence of malignant disease and mortality were also evaluated. Despite this, even larger cohorts should be evaluated, in order to deeply analyze tumor behavior, especially in those neoplasms with lower prevalence and previously known sex-related differences.

## Conclusions

Taken together, our results reveal that cortisol secretion in patients with AIs does not affect the prevalence of cancer, but it is associated with a worst clinical outcome and mortality. Additionally, its effect seems to be tumor specific and affects therapeutic response in some types of cancer. Further studies in larger cohorts that include specific comparison between mild autonomous cortisol secretion and overt Cushing syndrome should be performed in order to validate and enlarge the results of this study.

## Data availability statement

The original contributions presented in the study are included in the article/supplementary material. Further inquiries can be directed to the corresponding authors.

## Ethics statement

The studies involving humans were approved by Ramon y Cajal Ethics Committee. The studies were conducted in accordance with the local legislation and institutional requirements. The participants provided their written informed consent to participate in this study.

## Author contributions

AH-M: Conceptualization, Data curation, Formal Analysis, Investigation, Supervision, Writing – original draft, Writing – review & editing. ÁR: Formal Analysis, Investigation, Writing – original draft, Writing – review & editing. EP: Investigation, Writing – original draft, Writing – review & editing. CI: Investigation, Writing – original draft, Writing – review & editing. PP: Investigation, Writing – original draft, Writing – review & editing. PR: Writing – original draft, Writing – review & editing. CL: Investigation, Writing – original draft, Writing – review & editing. MA-C: Investigation, Supervision, Writing – original draft, Writing – review & editing.
